# Mirtazapine has a therapeutic potency in 1-methyl-4-phenyl-1,2,3,6-tetrahydropyridine (MPTP)-induced mice model of Parkinson’s disease

**DOI:** 10.1186/1471-2202-15-79

**Published:** 2014-06-25

**Authors:** Naoto Kadoguchi, Shinji Okabe, Yukio Yamamura, Misaki Shono, Tatsuya Fukano, Akie Tanabe, Hironori Yokoyama, Jiro Kasahara

**Affiliations:** 1 Department of Neurobiology and Therapeutics, Institute of Health Bioscience, Graduate School and Faculty of Pharmaceutical Sciences, The University of Tokushima, 1-78, Shoumachi, Tokushima 770-8505, Japan

**Keywords:** 1-methyl-4-phenyl-1,2,3,6-tetrahydropyridine (MPTP), Parkinson’s disease, Mirtazapine, Noradrenergic and specific serotonergic antidepressant (NaSSA), Serotonin (5-hydroxytryptamine, 5-HT), Dopamine

## Abstract

**Background:**

Mirtazapine, a noradrenergic and specific serotonergic antidepressant (NaSSA), shows multiple pharmacological actions such as inhibiting presynaptic α_2_ noradrenaline receptor (NAR) and selectively activating 5-hydroxytriptamine (5-HT) 1A receptor (5-HT_1A_R). Mirtazapine was also reported to increase dopamine release in the cortical neurons with 5-HT dependent manner. To examine whether mirtazapine has a therapeutic potency in Parkinson’s disease (PD), we examined this compound in 1-methyl-4-phenyl-1,2,3,6-tetrahydropyridine (MPTP)-treated mice model of PD.

**Results:**

Male C57BL/6 mice were subjected to MPTP treatment to establish a PD model. Mirtazapine was administered once a day for 3 days after MPTP treatment. MPTP-induced motor dysfunction, assessed by beam-walking and rota-rod tests, was significantly improved by administration of mirtazapine. Biochemical examinations by high performance liquid chromatography and western blot analysis suggested mirtazapine facilitated utilization of dopamine by increasing turnover and protein expression of transporters, without affecting on neurodegenerative process by MPTP. These therapeutic effects of mirtazapine were reduced by administration of WAY100635, an inhibitor for 5HT_1A_R, or of clonidine, a selective agonist for α_2_-NAR, or of prazosin, an inhibitor for α_1_-NAR, respectively.

**Conclusion:**

Our results showed mirtazapine had a therapeutic potency against PD in a mouse model. Because PD patients sometimes show depression together, it will be a useful drug for a future PD treatment.

## Background

Parkinson’s disease (PD) is a progressive, age-related, neurodegenerative disorder characterized by bradykinesia, resting tremor and gait disturbance. The major pathological basis of PD is the death of dopaminergic neurons in the substantia nigra pars compacta (SNc) and the degeneration of their nerve terminals in striatum [1]. It has been proposed that clinical signs of PD appear at the point when dopaminergic neuronal cell loss exceeds a critical threshold: 70 – 80% of striatal nerve terminals and 50 – 60% of the SNc perikaryons [2,3]. As a pharmaceutical treatment, ʟ-3,4-dihydroxyphenylalanine (ʟ-dopa), supplying the precursor of dopamine (DA), is the most commonly applied and alleviates major symptom of PD. For over 40 years, treatment with ʟ-dopa combined with an inhibitor for peripheral aromatic ʟ-amino acid decarboxylase (AADC) such as carbidopa had been established as a gold standard for PD treatment [4,5]. However, long-term treatment with ʟ-dopa is often complicated by the development of adverse effects such as drug-induced dyskinesia [6]. There have been additional anti-parkinsonian drugs, such as dopamine agonists mostly targeted on D_2_ class receptor, but their pharmaceutical potencies are not drastically better than that of ʟ-dopa. It is essential to develop novel drugs which can support therapeutic effects of ʟ-dopa with delaying expression of its side effects, or even solely effective against PD symptoms.

Serotonergic neurons play an important role in modulating extrapyramidal motor disorders such as PD and drug-induced parkinsonism [7,8]. Some studies showed administration of agonists for 1A subtype of serotonin/5-hydroxytriptamine receptor (5-HT_1A_R, e.g., 8-hydroxy-2-(di-n-propylamino)tertraline and tandospirone) significantly improved various types of extrapyramidal symptoms including antipsychotic-induced bradykinesia and catalepsy, and neurotoxin-induced bradykinesia [9-11]. Therefore, the central serotonergic system is thought to be one of a favorable drug target for the treatment of PD. It is a well-known fact that serotonergic as well as noradrenergic system is a major target of antidepressants such as selective serotonin-reuptake inhibitor (SSRI) fluoxetine and fluvoxamine. Recently, a novel antidepressant mirtazapine has been developed and is now approved in many countries for clinical treatment of major depression [12]. Mirtazapine is categorized into a noradrenergic and specific serotonergic antidepressant (NaSSA), showing multiple pharmacological actions such as inhibiting presynaptic α_2_ noradrenaline receptor (NAR) and selectively activating 5-HT_1A_R [13,14]. Mirtazapine has higher antidepressant effects than placebo or trazodone, which is equivalent to the effect of tricyclic antidepressant (TCA) such as clomipramine and amitriptyline [15-17]. Compared to SSRI, mirtazapine showed an earlier onset of antidepressant effects [18]. Further, the side effects of mirtazapine are reported to be lower than those of SSRI or TCA [19].

In 2004, Nakayama et al. reported mirtazapine increased DA release in the medial prefrontal cortex (mPFC) of rats with activating 5-HT_1A_R [20]. They reported that 8 or 16 mg/kg of mirtazapine increased DA release with dose-dependent manner, and an inhibitor of 5-HT_1A_R WAY100635 significantly decreased the mirtazapine-induced increase of DA release. We hypothesized mirtazapine may be effective on PD if the same mechanism as this 5-HT-dependent increase of DA release existed in the nigro-striatal dopaminergic system, too. In fact with related to PD, some studies reported the clinical efficacy of mirtazapine on Parkinsonian tremor in human [21,22]. However, little is known about the therapeutic effect of mirtazapine for motor dysfunctions other than tremor in PD. Therefore, in this study, we examined the effect of mirtazapine in mice treated with the neurotoxin 1-methyl-4-phenyl-1,2,3,6-tetrahydropyridine (MPTP), one of the typical animal models of PD [23,24].

## Results

### Effect of mirtazapine on motor dysfunctions induced by MPTP

We first assessed effect of mirtazapine on motor dysfunctions induced by MPTP using two different behavioral paradigms: the beam-walking and rota-rod tests.

We chose two doses of mirtazapine (4 and 16 mg/kg) in this study based on the previous study by Nakayama et al. in 2004 [20] in which they showed 4, 8 and 16 mg/kg of mirtazapine produced a dose-dependent increase in extracellular DA levels in mPFC of freely moving rats. Thus we examined minimal (4 mg/kg) and maximal (16 mg/kg) doses in mice.

**Figure 1:**
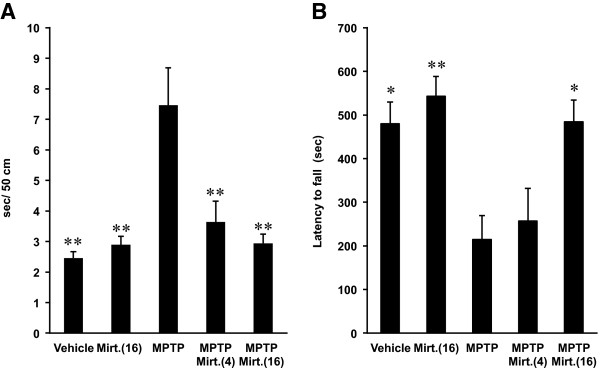
**Effects of mirtazapine on MPTP-induced motor dysfunctions in mice using beam-walking test and rota-rod test. (A)** Beam-walking test: Vertical axis shows the periods required to traverse 50 cm of the beam. **(B)** Rota-rod test: Vertical axis shows the latency to fall from the rotating rod after the mice were placed on it. Values are expressed as means ± SEM, n = 9–10 mice/group. Mirt.(4), mirtazapine 4 mg/kg; Mirt.(16), mirtazapine 16 mg/kg. Statistical significance was evaluated by one-way ANOVA followed by Scheffe test (*F*_(A) 4,44_ = 9.803, *F*_(B) 4,44_ = 7.341, *P < 0.05, **P < 0.01 compared with MPTP-treated group).

In beam-walking test, MPTP-treated mice showed a significantly prolonged duration to traverse a distance of 50 cm than that of the vehicle-treated mice (Figure 1A; *F*_(A) 4,44_ = 9.803, *P* < 0.01, ANOVA). In contrast, mirtazapine significantly improved the MPTP-induced prolongation of the traversal duration when it was treated after MPTP with both 4 and 16 mg/kg doses (Figure 1A; *P* < 0.01, ANOVA), although it did not affect when solely applied compared to the vehicle-treated mice (Figure 1A, *P* > 0.05, ANOVA).

With rota-rod test, vehicle-treated mice usually remained on the rotating rod for approximately 400–600 sec. As shown in Figure 1B, MPTP-treatment significantly decreased the latency to fall from the rod when compared to vehicle-treated mice (*F*_(B) 4,44_ = 7.341, *P* < 0.05, ANOVA). On the other hand, administration of 16 mg/kg of mirtazapine after MPTP significantly recovered the latency to the level of vehicle-treated group (Figure 1B; *P* < 0.05, ANOVA), with no effect when solely applied.

### Effect of mirtazapine on the striatal DA, DOPAC, HVA and turnover rate

**Figure 2:**
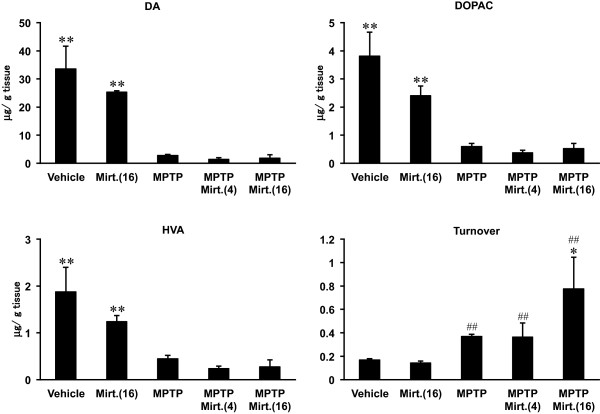
**Effects of mirtazapine on the striatal dopamine, DOPAC and HVA contents.** Values are expressed as means ± SEM, n = 5 mice/group. Mirt.(4), mirtazapine 4 mg/kg; Mirt.(16), mirtazapine 16 mg/kg. Statistical significance was evaluated by one-way ANOVA followed by Student-Newman-Keuls test (*F*_(DA) 4,20_ = 15.423, *F*_(DOPAC) 4,20_ = 10.767, *F*_(HVA) 4,20_ = 6.643, *F*_(Turnover) 4,20_ = 4.951, *P < 0.05, **P < 0.01 compared with MPTP-treated group) or by Student’s *t*-test (^**##**^*P* < 0.01 compared with vehicle group).

Using HPLC, we quantified the striatal DA and its metabolites DOPAC (3,4-dihydroxyphenylacetic acid) and HVA (homovanillic acid) with calculating turnover rate. As shown in Figure 2, administrations of MPTP produced marked depletion of DA, DOPAC and HVA in striatum (*F*_(DA) 4,20_ = 15.423, *F*_(DOPAC) 4,20_ = 10.767, *F*_(HVA) 4,20_ = 6.643, *P* < 0.01, ANOVA), as was reported previously [25,26]. DA turnover, calculated by (DOPAC + HVA)/DA [26], was increased significantly by MPTP treatment compared with vehicle (*P* < 0.01, Student’s *t*-test). Mirtazapine, when solely applied with 16 mg/kg, showed no significant alterations on them when compared with vehicle-treated group (Figure 2; *P* > 0.05, ANOVA). Furthermore, administrations of mirtazapine after MPTP treatment also showed no significant changes on them (Figure 2; *P* > 0.05, ANOVA) both with 4 and 16 mg/kg. However, DA turnover was significantly increased by 16 mg/kg of mirtazapine after MPTP treatment when compared with vehicle or MPTP alone (Figure 2; *F*_(Turnover) 4,20_ = 4.951, *P* < 0.05, ANOVA), although it did not affect the basal turnover when solely applied with 16 mg/kg (Figure 2; *P* > 0.05, ANOVA).

### Effect of mirtazapine on the striatal TH, DAT and VMAT2 protein expression

**Figure 3:**
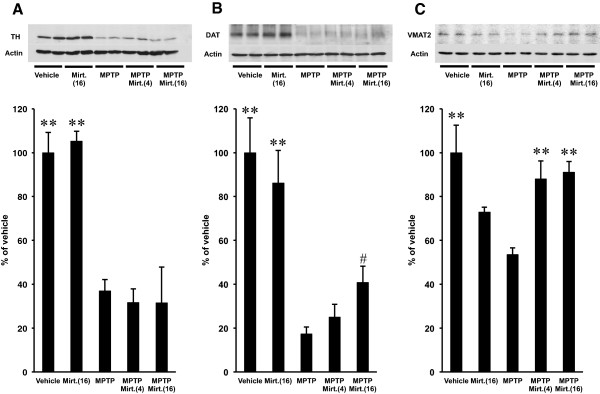
**Effects of mirtazapine on the striatal TH, DAT and VMAT2 protein expression examined by western blot analysis. (A)**, **(B)** and **(C)**, representative membrane image (upper panels) and the densitometric analysis of the positive bands (lower graphs) of TH **(A)**, DAT **(B)** and VMAT2 **(C)**. In **(A)**, **(B)** and **(C)**, actin protein was used as an internal control. Expression of TH, DAT and VMAT2 proteins are expressed as % of vehicle (means ± SEM), n = 4–5 mice/group. Mirt.(4), mirtazapine 4 mg/kg; Mirt.(16), mirtazapine 16 mg/kg. Statistical significance was evaluated by one-way ANOVA followed by Scheffe test (*F*_(TH) 4,17_ = 16.115, *F*_(DAT) 4,17_ = 12.386, *F*_(VMAT2) 4,19_ = 6.711 **P < 0.01 compared with MPTP-treated group) or by Student’s *t*-test (^**#**^*P* < 0.05 compared with MPTP-treated group).

With western blot analysis, we examined protein expression of the dopaminergic markers tyrosine hydroxylase (TH), dopamine transporter (DAT) and vesicle monoamine transporter 2 (VMAT2) in striatum. Treatment with mirtazapine, when solely applied with 16 mg/kg, showed no significant effects on the striatal TH, DAT and VMAT2 protein expression of mice when compared to the vehicle-treated group (Figure 3A,B and C; *F*_(TH) 4,17_ = 16.115, *F*_(DAT) 4,17_ = 12.386, *F*_(VMAT2) 4,19_ = 6.711, *P* > 0.05, ANOVA), although VMAT2 expression showed a slight tendency of decrease with no significance. MPTP significantly decreased TH, DAT and VMAT2 protein expressions to 20 – 50% of the vehicle treated ones (Figure 3A, B and C; *P* < 0.01, ANOVA). Mirtazapine did not alter protein expression of TH when it was applied after MPTP (Figure 3A; *P* > 0.05, ANOVA), strongly suggesting it did not affect the process of neurodegeneration of the nigro-striatal dopaminergic neurons triggered by MPTP. In contrast, mirtazapine showed a dose-dependent tendency of recovery of DAT protein expression, and 16 mg/kg of mirtazapine showed significant increase of DAT when compared with MPTP-treated group (Figure 3B; *P* < 0.05, Student’s *t*-test). Furthermore, both 4 and 16 mg/kg of mirtazapine showed significant recovery of VMAT2 protein expression compared with MPTP-treated group (Figure 3C; *P* < 0.01, ANOVA).

### Antagonism of WAY100635 on the behavioral effects of mirtazapine

**Figure 4:**
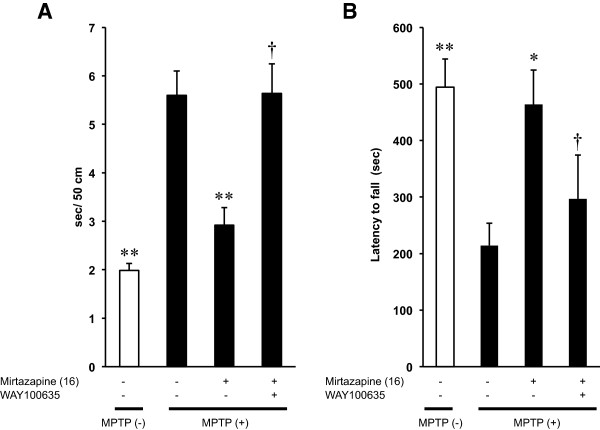
**Inhibition of the behavioral effects of mirtazapine by WAY100635 on beam-walking test and rota-rod test. (A)** Beam-walking test: Vertical axis shows the periods required to traverse 50 cm of the beam. **(B)** Rota-rod test: Vertical axis shows the latency to fall from the rotating rod after the mice were placed on it. Values are expressed as means ± SEM, n = 9–10 mice/group. Statistical significance was evaluated by one-way ANOVA followed by **(A)** Scheffe test and **(B)** Student-Newman-Keuls test. (*F*_(A) 3,35_ = 18.962, *F*_(B) 3,35_ = 5.488, *P < 0.05, **P < 0.01 compared with MPTP-treated group and ^†^*P* < 0.05 compared with MPTP + mirtazapine group).

One of the pharmacological effects of mirtazapine is a selective activation of 5-HT_1A_R with blocking both 5-HT_2_ and 5-HT_3_ receptors. To examine the involvement of this mechanism in the effects of mirtazapine, we tested WAY100635, a specific antagonist for 5-HT_1A_R, together with mirtazapine both on beam-walking and rota-rod tests. In both tests, the therapeutic effects of mirtazapine were almost completely cancelled by pre-treatment with 0.5 mg/kg of WAY100635 (Figure 4A and B; *F*_(A) 3,35_ = 18.962, *F*_(B) 3,35_ = 5.488, *P* < 0.01*,* ANOVA).

**Figure 5:**
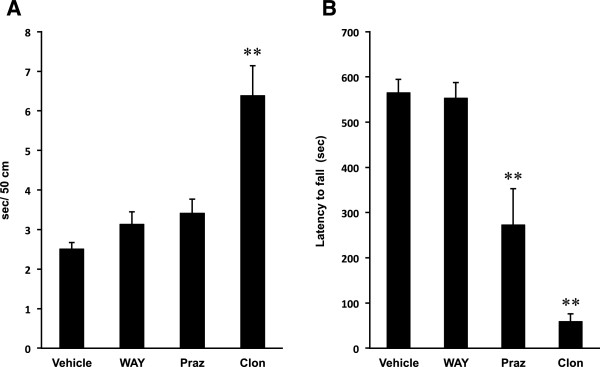
**Effects of WAY100635, prazosin or clonidine on basal motor activity of normal mice. (A)** Beam-walking test: Vertical axis shows the periods required to traverse 50 cm of the beam. **(B)** Rota-rod test: Vertical axis shows the latency to fall from the rotating rod after the mice were placed on it. Values are expressed as means ± SEM, n = 10 mice/group. WAY, WAY100635; Praz, prazosin; Clon, clonidine. Statistical significance was evaluated by one-way ANOVA followed by Scheffe test (*F*_(A) 3,36_ = 14.476, *F*_(B) 3,36_ = 27.092, **P < 0.01 compared with vehicle group).

We also examined the effects of WAY100635 on the basal activities of both tests, and it did not show any significant effects when compared with vehicle-treated group (Figure 5A and B; *F*_(A) 3,36_ = 14.476, *F*_(B) 3,36_ = 27.092, *P* > 0.05, ANOVA).

### Antagonism of prazosin or clonidine on the behavioral effects of mirtazapine

**Figure 6:**
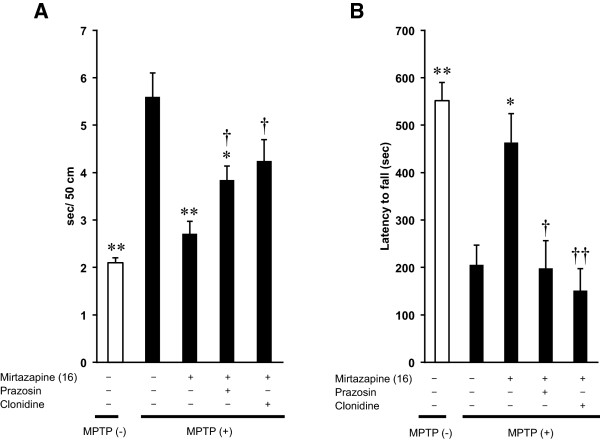
**Inhibition of the behavioral effects of mirtazapine by prazosin or by clonidine. (A)** Beam-walking test: Vertical axis shows the periods required to traverse 50 cm of the beam. **(B)** Rota-rod test: Vertical axis shows the latency to fall from the rotating rod after the mice were placed on it. Values are expressed as means ± SEM, n = 10 mice/group. Statistical significance was evaluated by one-way ANOVA followed by (A) Student-Newman-Keuls test and **(B)** Scheffe test (*F*_(A) 4,45_ = 15.060, *F*_(B) 4,45_ = 13.097, *P < 0.05, **P < 0.01 compared with MPTP-treated group and ^††^*P* < 0.01, ^†^*P* < 0.05 compared with MPTP + mirtazapine group).

Because noradrenergic system regulates both DA and 5-HT neurons [27-29] and α_2_-NAR is one of an inhibitory target of mirtazapine, we tested prazosin, an antagonist for α_1_-NAR, or clonidine, selective agonist for α_2_-NAR, together with mirtazapine both on beam-walking and rota-rod tests. As shown in Figure 6A and B, both of the noradrenergic drugs significantly reduced the effects of mirtazapine (Figure 6A and B; *F*_(A) 4,45_ = 15.060, *F*_(B) 4,45_ = 13.097*, P* < 0.01*,* ANOVA), although their effect in beam-walking test was incomplete when compared to that of WAY100635.

As we did in the previous section using WAY100635, we also examined both of the noradrenergic drugs on the basal behavioral activities of beam-walking and rota-rod tests. In beam-walking test, prazosin did not affect the periods for traversing 50 cm, although clonidine significantly increased it (Figure 5A; *P* < 0.01, ANOVA). In rota-rod test, both prazosin and clonidine significantly shortened the latency to fall from the rotating rod (Figure 5B; *P* < 0.01, ANOVA), suggesting some of the effects we have observed contain basal disturbance of these drugs on autonomic system.

### Antagonism of WAY100635, prazosin and clonidine on the biochemical effects of mirtazapine

We also examined the effects of WAY100635, prazosin and clonidine on the contents of the striatal DA and its metabolites with turnover rate of DA by HPLC both in the vehicle and MPTP-treated mice. As shown in Table 1, all of three drugs have no effects on basal DA, DOPAC and HVA contents, although prazosin and clonidine decreased basal DA turnover significantly when compared with vehicle-treated group (Table 1; *P* < 0.05 and *P* < 0.01, respectively, Student’s *t*-test). When these three drugs were administered prior to mirtazapine, all of them significantly reduced the increased DA turnover observed in MPTP + mirtazapine group (Table 1; *F*_(Turnover) 8,40_ = 4.232, *P <* 0.05, ANOVA).

**Table 1:** The effect of WAY100635, prazosin or clonidine on the striatal dopamine, DOPAC and HVA

	**Dopamine**	**DOPAC**	**HVA**	**Turnover rate**
Vehicle	19.49 ± 0.75**	1.62 ± 0.12**	1.03 ± 0.04**	0.14 ± 0.01
WAY100635 (0.05 mg/kg)	18.22 ± 1.21**	1.73 ± 0.09**	0.91 ± 0.04**	0.15 ± 0.01
prazosin (0.03 mg/kg)	20.27 ± 2.33**	1.51 ± 0.14**	0.79 ± 0.09**	0.12 ± 0.01^**#**^
clonidine (0.15 mg/kg)	22.76 ± 1.88**	1.47 ± 0.07**	0.84 ± 0.05**	0.10 ± 0.01^**##**^
MPTP	3.38 ± 0.35	0.51 ± 0.05	0.44 ± 0.04	0.28 ± 0.01^**##**^
MPTP + mirtazapine (16 mg/kg)	1.90 ± 1.21	0.38 ± 0.08	0.29 ± 0.06	0.78 ± 0.25*
MPTP + mirtazapine (16 mg/kg) + WAY100635 (0.05 mg/kg)	2.25 ± 0.47	0.40 ± 0.10	0.32 ± 0.08	0.33 ± 0.05^†^
MPTP + mirtazapine (16 mg/kg) + prazosin (0.03 mg/kg)	3.49 ± 0.48	0.56 ± 0.04	0.37 ± 0.01	0.28 ± 0.03^†^
MPTP + mirtazapine (16 mg/kg) + clonidine (0.15 mg/kg)	2.90 ± 0.68	0.58 ± 0.68	0.45 ± 0.07	0.31 ± 0.04^†^

The results are shown as the mean (μg/g tissue) ± SEM of 4–6 animals/group. Statistical significance was evaluated by Student-Newman-Keuls test (*F*_(DA) 8,40_ = 84.267, *F*_(DOPAC) 8,40_ = 32.896, *F*_(HVA) 8,40_ = 29.578, *F*_(Turnover) 8,40_ = 4.232, *P < 0.05,**P < 0.01 compared with MPTP-treated group and ^†^*P* < 0.05 compared with MPTP + mirtazapine group), or by Student’s *t*-test (^**#**^*P* < 0.05, ^**##**^*P* < 0.01 compared with vehicle group).

### RT-PCR detection of mRNA for the isoforms of noradrenaline and serotonin receptors

**Figure 7:**
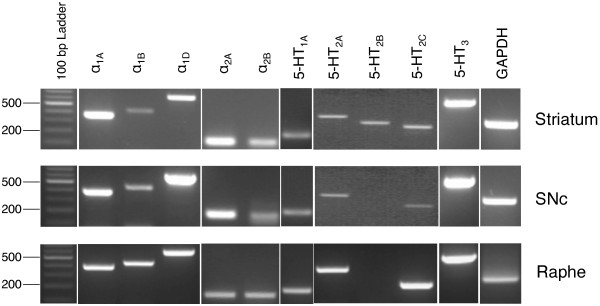
**RT-PCR detection of mRNAs for the isoforms of NA and 5-HT receptors.** RT-PCR was performed as described in Methods. PCR products were subjected to agarose gel electrophoresis, and the gel images with UV detection are shown.

To examine whether the known receptors, which could be affected with mirtazapine directly or for the targets of the inhibitors used in this study, are expressed in striatum, SNc and raphe nucleus, we performed RT-PCR. The specific primers used to detect mRNAs for the noradrenaline and 5-HT receptors, α_1A_, α_1B_, α_1D_, α_2A_, α_2B_, 5-HT_1A_, 5-HT_2A_, 5-HT_2B_, 5-HT_2C_ and 5-HT_3_ are written in Methods. As shown in Figure 7, α_1A_, α_1B_, α_1D_, α_2A_ and α_2B_ noradrenaline receptors were expressed in striatum, SNc and raphe. On the other hand, no 5-HT_2B_R transcript was detected in SNc and raphe, while 5-HT_1A_, 5-HT_2A_, 5-HT_2C_ and 5-HT_3_ receptors were detected in striatum, SNc and raphe (Figure 7).

## Discussion

In the present study, we found that treatment with mirtazapine in mice significantly improved MPTP-induced motor dysfunction. To our knowledge, this is the first report showing the therapeutic potency of an antidepressant mirtazapine against MPTP neurotoxicity in mice. Because MPTP mice are one of the most popular models for screening anti-PD agents [23-26,30], our results suggest possible use of mirtazapine as a PD therapeutics in clinical patients.

Our results of HPLC in Figure 2 and Table 1 showed MPTP increased DA turnover in striatum, and mirtazapine further elevated it. Similar results were reported previously by zonisamide, an anti-convulsant drug also effective on PD [26]. Increased DA turnover with MPTP treatment is thought as a compensatory effect exerted by the remained DA neurons under the neurotoxic condition [26,31], although significant behavioral deficits (Figure 1) suggested the compensatory effect was insufficient to keep the normal motor coordination. Further elevation of DA turnover with mirtazapine after MPTP treatment observed in this study led us to speculate mirtazapine facilitate utilization of DA, probably by increasing DA release, reuptake, degradation and/or recycling in the DA-depleted condition. Supporting this idea, in fact, reduced protein expression of DAT by MPTP was partially recovered, and that of VMAT2 was almost completely recovered to the normal level by mirtazapine (Figure 3B and C). The increase of these transporter proteins would reflect the increased DA release from the dopaminergic nerve terminals in striatum by mirtazapine [32,33]. In contrast, reduction of TH expression with MPTP was not rescued by mirtazapine (Figure 3A), suggesting it did not affect on the neurodegenerative process of MPTP.

**Figure 8:**
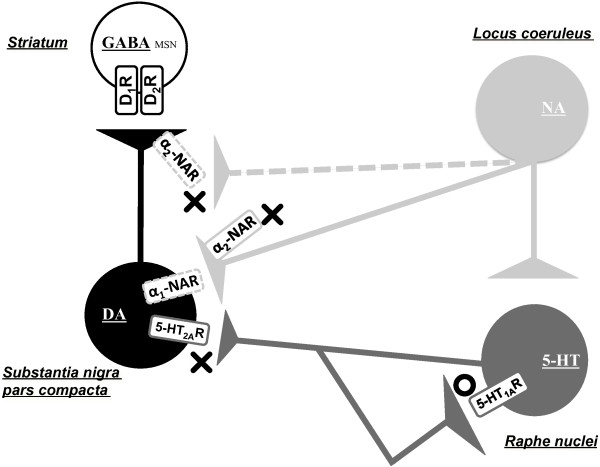
**Hypothetical mechanisms of therapeutic effects against PD by mirtazapine.** DA, dopamine, 5-HT, 5-hydroxytryptamine (serotonin); NA, noradrenaline; GABA, γ-aminobutylic acid; MSN, medial spiny neuron; D_1_R, dopamine D_1_ receptor; D_2_R, dopamine D_2_ receptor; α_1_-NAR, α_1_ noradrenaline receptor; α_2_-NAR, α_2_ noradrenaline receptor; 5-HT_1A_R, 5-HT_1A_ receptor: 5-HT_2_R, 5-HT_2_ receptor. Dashed lines indicate hypothetical innervation and receptors. The circle indicates a stimulatory effect by mirtazapine, while the crosses indicate an inhibitory effect by mirtazapine. See details in Discussion.

The effect of mirtazapine in our study was expressed specifically after the treatment of MPTP, and the sole treatment with mirtazapine did not alter the behavioral parameters (Figure 1) nor the striatal contents of DA and its metabolites with DA turnover (Figure 2 and Table 1), whereas the previous report of Nakayama et al. [20] showed increased DA release by acute and sole treatment of mirtazapine in mPFC of rats. The discrepancy is probably caused by following differences: schedule of drug administration, method and timing of sampling, method of analysis especially because the lack of real-time measurement of extracellular DA levels in our study. Nevertheless of the discrepancy, these results suggest increase of DA by mirtazapine in the rodent brain with a short-term administration. Other study, however, reported that two-week administration of mirtazapine completely antagonized the stress-induced increase in dopamine release in the prefrontal cortex [34]. It may be possible that the pharmacological action of miratazapine is different depending on the periods of administration and the amount of DA at the targeted synapses. Further examination using microdialysis in striatum measuring extracellular DA, NA and 5-HT with their metabolites will explore more precise mechanisms of both acute and chronic action of mirtazapine including the hypothesis shown in Figure 8.

As mentioned in Introduction, mirtazapine is categorized into NaSSA, inhibiting pre-synaptic α_2_-NAR specifically. Mirtazapine also inhibits 5-HT_2_ and 5-HT_3_ receptors which in turn selectively activate 5-HT_1_R. It enhances, therefore, the release of noradrenaline and 5-HT_1A_R-mediated serotonergic transmission [35]. Based on these pharmacological properties of mirtazapine and from our results of the experiments using NAR- and 5-HTR-related reagents, we illustrated a hypothetical mechanism of mirtazapine action on PD-related nigro-striatal dopaminergic system with serotonergic and noradrenergic systems (Figure 8). WAY100635, a selective inhibitor for 5-HT_1A_R, clearly cancelled the therapeutic effects of mirtazapine on MPTP-induced neurotoxicity without affecting the basal behavioral or the biochemical parameters (Figures 4 and 5, Table 1), strongly suggest the involvement of this receptor for the effects of mirtazapine. From the dorsal raphe nuclei, 5-HT neurons innervate directly to the nigral DA neurons to inhibit the firing of them with 5-HT_2A_R-dependent manner [28,36]. Inhibition of this 5-HT_2A_R by mirtazapine results in increase of DA release. Further, the recurrent innervation of 5-HT neurons via 5-HT_1A_R in raphe negatively controls cell firing and release of 5-HT [37]. Thus, activation of 5-HT_1A_R by mirtazapine can reduce 5-HT release, and it also results in the increased DA release. In fact, our RT-PCR results showed expression of both 5-HT_2A_R mRNA in SNc and 5-HT_1A_R in raphe (Figure 7). Recently, it has been reported that 5-HT_2A_R antagonists M100907 improved motor impairments in the MPTP-induced mouse model of PD [38]. Other studies also revealed that stimulation of 5-HT_1A_R recovered the motor disorders caused by lesions of DA neurons or DA depletion [39-42]. Thus, both 5-HT_1A_R and 5-HT_2A_R are attractive targets as novel PD therapeutics. α_2_-NAR is another inhibitory target of mirtazapine. In fact, clonidine, a selective agonist for this receptor reduced the effects of mirtazapine, although it also affected basal activity of mice and striatal DA turnover (Figures 6 and 5, Table 1), probably of its inhibitory effect on peripheral sympathetic system. Noradrenaline inhibits the neurotransmitter release via presynaptic α_2_-NAR, and mirtazapine increases neurotransmitter release by inhibiting this receptor [13,14]. Although precise mechanism is still unknown, we speculate this receptor could also function as presynaptic hetero receptor on DA neuron to regulate DA release negatively, the same as presynaptic autoreceptor on noradrenergic nerve terminal (Figure 8). Actually we detected α_2A_ and α_2B_ mRNA isoforms both in striatum and SNc (Figure 7). Prazosin, an antagonist for α_1_-NAR, also showed similar effects as clonidine. We suppose noradrenergic input on DA would activate DA neuron via α_1_-NAR in SNc as illustrated in Figure 8, since similar stimulatory mechanism was reported in 5-HT and other neurons [43]. However, we could not clearly discriminate peripheral and central effects of the noradrenergic drugs in this study. It will be difficult to use clonidine (0.15 mg/kg) and prazosin (0.03 mg/kg) to address the mechanism for mirtazapine effect in this mouse model with a systemic administration. Precise mechanism of the effect of mirtazapine through noradrenergic system should be examined, with applying more specific inhibitors directly into specific brain regions, for example.

It is a well known fact that long-term treatment of ʟ-dopa in PD patients causes various adverse side effects such as wearing-off, dyskinesia, psychiatric symptoms, and so on [44,45]. A recent study has demonstrated that treatment with a SSRI fluoxetine significantly suppressed ʟ-dopa-induced rotational behavior in 6-hydroxydopamine (6-OHDA)-treated rats with 5-HT_1A_R-dependent manner [46]. Furthermore, a selective α_2_-NAR antagonist fipamezole reduced ʟ-dopa-induced dyskinesias in MPTP-treated monkeys [47]. These observations suggest that NaSSA mirtazapine could be a possible novel therapeutic drug for PD, particularly in regard to avoiding the adverse side effects of ʟ-dopa. Depression in PD patients is the most common psychiatric disturbance [48], and SSRIs are now often used for the treatment [49]. Recently, NaSSA is also used and shown to be effective in the treatment of depression in PD patients as well as SSRI [48,50]. However, the effect of NaSSA on motor dysfunction in PD patients is still unknown. Together with these reports and our results in this study, it is highly expected that mirtazapine has dual therapeutic effects both on depression and PD in humans. Our study here and further detailed examinations will open the next door of clinical trial to examine mirtazapine on PD.

## Conclusion

Our present study provides the first evidence that mirtazapine has a therapeutic potency against MPTP neurotoxicity in mice. Because PD patients sometimes show depression together, it is highly expected that mirtazapine has dual therapeutic effects both on depression and PD in humans.

## Methods

### Experimental animals

Male C57BL/6 mice (Nihon SLC Co., Shizuoka, Japan), 8 weeks of age, were used in this study. The animals were housed in a controlled environment (23 ± 1°C, 50 ± 5% humidity) and were allowed food and tap water *ad libitum*. The room lights were on between 8:00 and 20:00. All handlings and procedures of animal experiments were performed in accordance with the National Institute of Health guide for the care and use of Laboratory animals (NIH Publications No. 8023, revised 1978), and approved by the Committee for Animal Experiments of the University of Tokushima (#10138).

### Drug treatments

Mice were injected with 20 mg/kg of MPTP (Sigma-Aldrich, St. Louis, MO, USA) or saline intraperitoneally (i.p.) every 2 hr for a total of four injections, resulting in a cumulative dose of 80 mg/kg, as described previously [51]. Mirtazapine (generously provided by Meiji Seika Pharma Co., Ltd., Japan) was dissolved in 0.5% carboxymethylcellulose, and was applied once a day with 4 or 16 mg/kg i.p. started from 1 hr after the final MPTP treatment for 4 days. WAY100635 (0.5 mg/kg, Sigma-Aldrich, St. Louis, MO, USA), prazosin (0.03 mg/kg, Sigma-Aldrich, St. Louis, MO, USA) and clonidine (0.15 mg/kg, Sigma-Aldrich, St. Louis, MO, USA) were dissolved in saline, and each of them was administered 1 hr before treating with mirtazapine.

### Behavioral testing

Three days after MPTP or saline treatment, behavioral tests were performed 1 hr after the final treatment with mirtazapine or vehicle. For the behavioral analysis, we examined with two different experimental paradigms.

#### Beam-walking test

The apparatus used in this experiment was a modification of that used by Allbutt and Henderson [52]. Before MPTP treatment, mice were trained to transverse a wooden round beam with 12 mm diameter, 80 cm length, suspended 55 cm above the floor, in two consecutive trains with 3 hr intervals each day for 3 days. Three days post-MPTP or saline treatment, mice were subjected to trials on the beam with 8 mm rather than 12 mm diameter during which they were video-recorded. The time to reach a distance of 50 cm was measured.

#### Rota rod test

The Rota rod treadmill (Constant Speed Model, Ugo Basile, Varese, Italy) consists of a plastic rod, 6 cm in diameter and 36 cm long, with a non-slippery surface 20 cm above the base (trip plate). This rod is divided into five equal sections by six discs (25 cm in diameter), which enables five mice to walk on the rod at the same time. In the present study, rotor mode with a constant speed was used. All the mice used were subjected to one training session a day for 3 consecutive days before MPTP treatment with 20 rpm for 10 min. At the trial session performed 3 days after MPTP treatment, the latency to fall of the animals from the rotating rod (32 rpm for 10 min) after they were placed on it was recorded as the performance time, as described previously [25].

### Quantification of DA and its metabolites

The mice were killed by cervical dislocation 3 days after the final treatment with saline or MPTP. The striatum were rapidly dissected out on ice and sonicated in ice-cold 50 nM perchloric acid containing 1 μg/ml isoproterenol as an internal standard. DA, DOPAC and HVA were quantified by HPLC with an electrochemical detector (ECD) (Eicom, Kyoto, Japan). Concentrations of dopamine and its metabolites were expressed as μg/g tissue weight, as described previously [53,54].

### Western blot analysis

The striatal tissues were homogenized in (50 mM Tris–HCl, pH 7.5, 0.5 M NaCl, 0.5% Triton X-100, 10 mM EDTA, 4 mM EGTA, 1 mM Na_3_VO_4_, 30 mM Na_4_P_2_O_7_, 50 mM NaF, 0.1 mM leupeptin, 0.075 mM pepstatin A, 0.05 mg/ml trypsin inhibitor, 1 mM phenylmethanesulfonyl fluoride, 100 nM calyculin A, and 1 mM dithiothreitol) using a microtube homogenizer. Insoluble materials were removed by centrifugation at 15,000 rpm (CT15RE, HITACHI, Ibaragi, Japan) for 10 min. The supernatants were mixed with Laemmli's sample buffer (final concentrations, 63 mM Tris–HCl, pH 6.8, 2% SDS, 5% β-mercaptoethanol, 2.5% glycerol, and 0.0083% bromphenol blue) and boiled for 5 min. Protein concentrations of the sample were determined using the Bradford protein assay. Ten micrograms of protein from each sample were separated on 12% sodium dodecyl sulfate-polyacrylamide gel electrophoresis (SDS-PAGE) gel using constant current. Separated proteins were transferred to polyvinylidene difluoride (PVDF) membranes at 75 V for 1.5 hr using a Trans Blot Cell (Bio-Rad, CA, USA). The membranes were incubated for 1 h at room temperature with Tris-buffered saline containing 0.1% Tween 20 (TBST) and 0.5% skim milk, followed by overnight incubation at 4°C with desired primary antibodies. The anti-TH antibody (1:1000, Chemicon International, Inc., Temecula, CA, USA) and anti-DAT antibody (1:1000, Chemicon International Inc., Temecula, CA, USA) as a marker of dopaminergic neurons were used. The anti-VMAT2 antibody (1:500, Santa Cruz Biotechnology, CA, USA) as a maker of presynaptic components was used. Membranes were washed six times for 5 min each at room temperature and incubated with horseradish peroxidase-conjugated secondary antibody in TBST for 1 hr. Immunoreactive bands were visualized by enhanced chemiluminescent autoradiography (ECL Kit, GE healthcare, Buckinghamshire, UK), according to manufacturer’s instructions. Actin antibody (Sigma, Saint Louis, MO, USA) was used as a house keeping protein to confirm that equal amounts of protein were loaded in each line. Optical densities were determined using a computerized image analysis system (Dolphin-DOC, Kurabo, Osaka, Japan), as described previously [25,55].

### RNA isolation and RT-PCR

Total RNA from striatum, SNc or raphe was purified using RNAiso plus (Takara Bio, Tokyo, Japan) according to manufacturer’s protocol. Reverse transcription from RNA to cDNA was performed using M-MLV reverse transcriptase and other supplements (Promega, Madison, WI, USA). Polymerase chain reaction (PCR) with GoTaq Green (Promega, Madison, WI, USA) was performed with using glyceraldehyde phosphate dehydrogenase (GAPDH) as an internal control. The following primers for mouse noradrenaline and 5-HT receptors with GAPDH were used (name, accession number, forward (F) or reverse (R) primer sequence, product length):

α_1A_, NM_07417, (F) CGACAAGTCAGACTCAGAGCAAGTGA,

(R) TGTAGCCCAGGGCATGCTTGGAAGAC, 403 bp;

α_1B_, NM_007416, (F) TTTCATGAGGACACCCTCAGCAGTACC,

(R) CTGCCACTGTCATCCAGAGAGTCC, 451 bp;

α_1D_, NM_013460, (F) CGCCAAAGGAAATCCAGGGACAC,

(R): CAGAGCGGAACTTATGGGACAGG, 616 bp;

α_2A_, NM_013461, (F) TTCTTTTTCACCTACACGCTCA,

(R) TGTAGATAACAGGGTTCAGCGA, 121 bp;

α_2B_, NM_009633, (F) ACCTTCCCTTGCTGACTGTACT,

(R) TGGGAGGGAGGTATTCTAATCA, 111 bp;

5HT_1A_, NM_008303, (F) TGCTCATGCTGGTCCTCTAT,

(R) TCTCAGCACTGCGCCTGC, 179 bp;

5HT_2A_, NM_172812, (F) AAGCCTCGAACTGGACAATTGATG,

(R) TGATTTTCAGGAAGGCTTTGGTT, 477 bp;

5HT_2B_, NM_008311, (F) CTCGGGGGTGAATCCTCTGA,

(R) CCTGCTCATCACCTCTCTCAG, 366 bp;

5HT_2C_, NM_008312, (F) CAGATCAGAAGCCACGTCGA,

(R) GGCTTATAATCGCAGCGCAA, 318 bp;

5HT_3_, NM_013561, (F) GCCACCCAGGCCCGAGATAC,

(R) GCTCCCACTCGCCCTGATTT, 564 bp;

GAPDH, M32599, (F) GCCAAAGGTCATCCATGACAACTTTG,

(R) CATTGGGGGTAGGAACACGGAAGGC, 237 bp;

All of the PCR reactions were started with heating 95°C for 2 min, followed by the repeated cycle of 95°C for 30 sec, 57 - 61°C for 45 sec and 72°C for 1 min, with additional 72°C for 10 min. After repeated thermal reactions for 30 cycles, the PCR products were separated by gel electrophoresis with 1% agarose containing 5 μg/mL of ethidium bromide.

### Statistical analysis

All values were expressed as the means ± SEM and statistical significance was evaluated by one-way analysis of variance (ANOVA) followed by Scheffe test, Student-Newman-Keuls test or Student’s *t*-test (Stat View version 5.0, SAS Institute Inc., USA). Statistical differences were considered with significance at *P* < 0.05.

## Abbreviations

5-HT: 5-hydroxytriptamine; 5-HT1AR: 5-hydroxytriptamine 1A receptor; AADC: ʟ-amino acid decarboxylase; DA: Dopamine; DAT: Dopamine transporter; DOPAC: 3,4-dihydroxyphenylacetic acid; ECD: Electrochemical detector; GAPDH: Glyceraldehyde phosphate dehydrogenase; HPLC: High performance liquid chromatography; HVA: Homovanillic acid; ʟ-dopa: ʟ-3,4-dihydroxyphenylalanine; mPFC: Medial prefrontal cortex; MPTP: 1-methyl-4-phenyl-1,2,3,6-tetrahydropyridine; NAR: Noradrenaline receptor; NaSSA: Noradrenergic and specific serotonergic antidepressant; PCR: Polymerase chain reaction; PD: Parkinson’s disease; PVDF: Polyvinylidene difluoride; SDS-PAGE: Sodium dodecyl sulfate-polyacrylamide gel electrophoresis; SNc: Substantia nigra pars compacta; SSRI: Serotonin-reuptake inhibitor; TBST: Tris-buffered saline containing 0.1% Tween 20; TCA: Tricyclic antidepressant; TH: Tyrosine hydroxylase; VMAT2: Vesicular monoamine transporter 2.

## Competing interests

The authors declare that they have no competing interests.

## Authors’ contributions

NK and SO equally contributed to this work, performed drug treatment, behavioral testing, HPLC, western blot, statistical analysis, and wrote a part of the manuscript. YY, AT and HY performed a part of behavioral testing and data analysis. MS and TF performed RNA isolation and RT-PCR. JK conceived, designed and supervised all the experiments, and prepared the manuscript. All the authors read and approved the final manuscript.
